# Primary thyroid lymphoma: a case series

**DOI:** 10.1186/s13256-024-04434-1

**Published:** 2024-02-24

**Authors:** Saida Sakhri, Ines Zemni, Mohamed Ali Ayadi, Salma Kamoun, Riadh Chargui, Tarek Ben Dhiab

**Affiliations:** 1 Department of Surgical Oncology, Salah Azaiez Institute, Faculty of Medicine of Tunis, University of Tunis El Manar, Tunis, Tunisia; 2 LMBA (LR03ES03), Sciences Faculty of Tunis, University Tunis El Manar, Tunis, Tunisia; 3 Department of Pathology, Salah Azaïz Institute, Faculty of Medicine, University Tunis El Manar, Tunis, Tunisia

**Keywords:** Thyroid, Lymphoma, Surgery, Chemotherapy, Prognosis

## Abstract

**Introduction:**

Primary Thyroid Lymphoma (PTL) is defined as lymphoma involving the thyroid gland alone or the thyroid gland and adjacent neck lymph nodes without contiguous spread or distant metastases at the time of diagnosis. Most thyroid lymphomas are B cell lymphomas, and 98% of all PTL cases are non-Hodgkin’s lymphoma. It is a rare disease accounting for around 5% of the thyroid neoplasms and 2% of extranodal lymphomas. If properly diagnosed and treated, the prognosis is favorable.

**Case presentation:**

Five cases (three men and two women) of PTL were diagnosed and treated in our institute between January 2005 and September 2019. These are 5 cases of Caucasian origin. The mean age was 76.2 (range: 63–95 years); one patient had associated hypothyroid. One patient had a medical history of breast cancer; one was hypothyroid, and four were euthyroid at the diagnosis. In 4 of these patients, PTL started with compressive symptoms. No patients underwent fine needle aspiration cytology (FNAC) or biopsy for the diagnostic only. In sonography, two cases showed bilateral nodules with goiter; in the three cases it showed nodules in the lobe and isthmus. Technetium-99m scintigraphy was performed on only two patients. Bone Marrow Biopsy (BMB) showed normal cellularity in 4 cases and only one case showed tumor cells. LDH levels were increased in all cases. The extension was evaluated in all patients with cervical and thoracic CT scans, Bone Marrow Biopsy (BMB), beta-2 microglobulin, and serum lactate dehydrogenase (LDH) levels. Three cases were staged as IE and two cases as IIE. Three patients underwent total thyroidectomy; two of them underwent cervical lymph node dissection. Two patients underwent lobectomy. All were diagnosed with lymphoma postoperatively and all were diffuse large B cell lymphoma (DLBCL). One patient completed treatment with R-CHOP (Rituximab, Cyclophosphamide, Doxorubicin, Vincristine, and Prednisone), and two cases received adjuvant chemo-radiotherapy (30 Gy). Two patients died immediately after surgery.

**Conclusion:**

PTL is a rare disease whose diagnosis should be considered in cases of rapidly growing goitres. Timely needle biopsy in suspected cases can avoid unnecessary surgery. Systemic treatment is required, depending on the stage of the tumour.

## Introduction

Primary Thyroid Lymphoma (PTL) is defined as lymphoma involving the thyroid gland alone or the thyroid gland and adjacent neck lymph nodes without contiguous spread or distant metastases at the time of diagnosis. The most common presentation is a rapidly growing painless mass in the neck, causing compression symptoms. PTL is most common in women in their sixth or seventh decade of life. The main risk factor is the presence of Hashimoto’s thyroiditis [3].

There is two distinct histological types: diffuse large B cell lymphoma (DLBCL) and mucosa-associated lymphoid tissue (MALT) lymphoma [1, 2]. Most thyroid lymphomas are B cell lymphomas, and 98% of all PTL cases are non-Hodgkin’s lymphoma. Diagnosis is made by fine needle aspiration (FNA), and cytology may be sufficient for diagnosis. Accurate diagnosis by cytology is important, as treatment depends on the lymphoma subtype [2].

It is a rare disease accounting for around 5% of thyroid neoplasms and 2% of extranodal lymphomas. The annual incidence of PTL is one or two cases per million. The prognosis is very good depending on the lymphoma subtype, with a 5-year survival rate of up to 45% [3]. The management and prognosis of PTL have changed with the advent of multimodal adjuvant therapy [4].

This study aimed to identify the clinical and pathological features of PTLs treated at a single institution.

## Case presentation

**Fig. 1:**
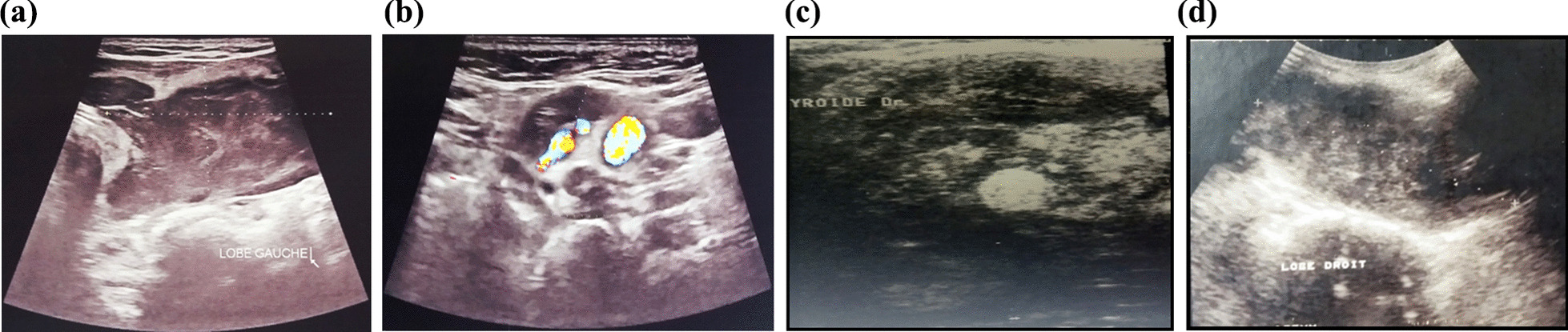
Cervical ultrasound findings **A** a multinodular goiter with a large hypoechoic left isthmo-lobar nodule with lobulated contours. **B** Cervical Doppler ultrasound showing a vascularized nodule. **C** Cervical ultrasound showing a heterogeneous, hyperechoic nodule in the right lobe, suspect. **D** The right lobe is enlarged and vascularized with irregular limit

Five cases (three men and two women) were diagnosed with PTL between 2005 and 2019. These are 5 cases of Caucasian origin. The mean age was 76.2 (range 63–95). Epidemiological and clinical characteristics of patients are presented in Table 1. On Ultrasonography themean diameter was 7.2 cm (Fig. 1). No patients underwent fine needle aspiration cytology (FNAC) or biopsy for purely the diagnostic purposes.

**Table 1:** Epidemiological and clinical characteristics of patients

Case	Gender	Age	Medical history	Symptoms	Physical examniation	Latencyperiod	Cervical ultrasound	FT4 /TSH	LDH
1	female	63	Breast cancer	dysphonia	Cervical mass measuring 7x7cm	3 months	multinodular goiter with a large hypoechoic left isthmo-lobar nodule with lobulated contours TIRADS 5 (Fig. 1A, B)	Normal	1xnormal
2	Male	79	-Asthma -diabetes -hypothyroid	-Dysphagia -Shortness of breath -goitre	-goitre -mass in the isthmus measuring 4 cm	6 months	Nodule in the isthmus hypechoic TIRADS 5	Hypothyroid	1xnormal
3	female	95	None	Dysphagia Dyspnea dysphonia	Mass fixed, measuring 12x8 cm	15 years	Nodule in the right lobe,with heterogeneous Structures (Fig. 1C) -cervical lymhadenopathy	Normal	7xnormal
4	Male	69	None	Dysphonia dysphagia	fixedMass, measuring 10 cm, -cervical lymhadenopathy	2 months	The right lobe is enlarged measuring 10.5X5.6X6 cm irregular limit, vascularized (Fig. 1D) - cervical lymhadenopathy TIRADS 5	Normal	3xnormal
5	Male	75	None	Dysphagia	Goiter Mass in the isthmus measuring 3 cm	6 months	Nodule on the isthmus and left lobe TIRADS 5	Normal	NA

**Fig. 2:**
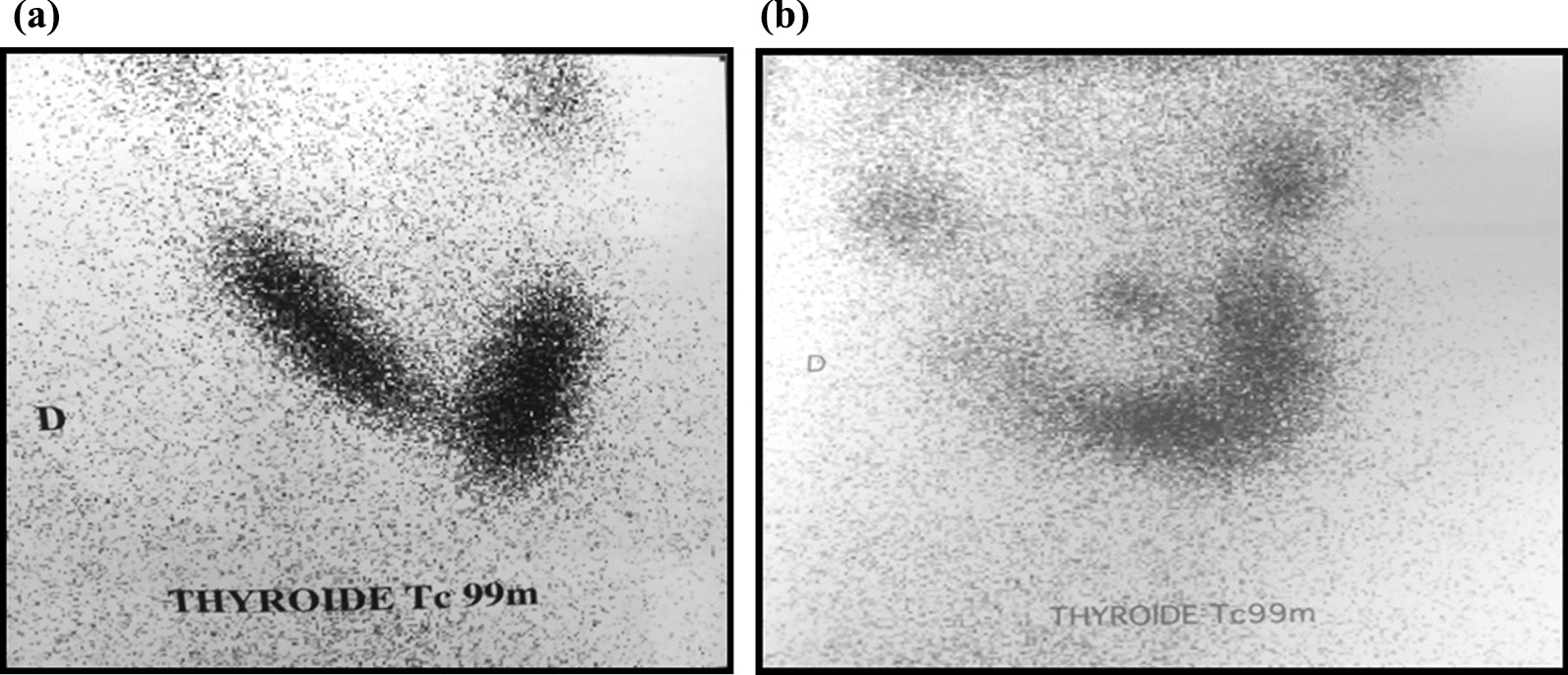
Tc 99 m Scintigraphy showing **A** a thyroid in place with hypertrophy of the right lobe. The left lobe is homogeneously fixed. **B** A heterogeneous fixation of the thyroid with multiple cold nodules

Technetium-99m scintigraphy was performed in only two patients (case1 and 4); in case 4, it showed enlargement of the lower portion of the right thyroid lobe (Fig. 2A); in the second case (case 1) it showed a heterogeneous fixation of the thyroid with multiple cold nodules (Fig. 2B).

Bone Marrow Biopsy (BMB) showed normal cellularity in 4 cases and tumour cells in only one (case4). Lactate dehydrogenase (LDH) levels were increased in all cases. Extension was assessed in all patients by cervical and thoracic CT scan, BMB, beta-2 microglobuline and serum lactate dehydrogenase (LDH) levels. According to the Ann-Arbor staging system, three cases were classified as stage IE, one as stage IIE and one as stage IV.

Three patients underwent total thyroidectomy, and two underwent cervical lymph node dissection. Two patients underwent lobectomy. Lymphoma was diagnosed in all patients after surgery, and in all cases it was DLBCL.

**Fig. 3:**
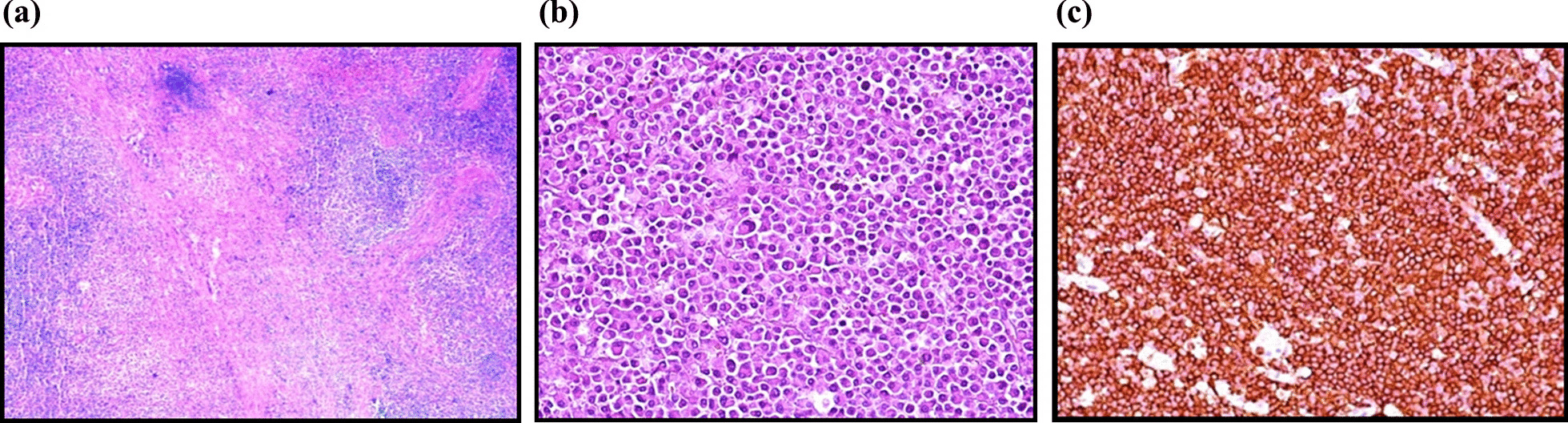
Histological findings. **A** Diffuse atypical lymphoid infiltration and interstitial small follicles consisting of thyrocytes (HEX10)**. B** Small B cell lymphomas with plasmacytic differentiation (HEX40). **C** Diffuse large B-cell Non-Hodgkin lymphoma in CD20 positive neoplastic cells (IHC)

Pathological examination revealed primary thyroid lymphoma. In addition, CD 20 was positive and CD 3, CD 45 RO, Bcl-6 and Bcl-2 were negative in Immunohistochemical (IHC) studies in all patients (Fig. 3). One patient completed treatment with R-CHOP (Rituximab, Cyclophosphamide, Doxorubicin, Vincristine and Prednisone) and two cases received chemotherapy used in combination with radiotherapy. Two patients died immediately after surgery due to respiratory distress (Table 2).

**Table 2:** Histology, treatment, and follow up data of patients

Patients	Histological type + IHC	Tumor size	Ki67	Clinical stage	Treatment	Disease specific survival
1	CD20 + CD3 − DLBCL	8x4x2 cm	NA	IIE	Lobo-isthmectomy + chemotherapy (CHOP) + ritiximab	26 months
2	CD79a + CD20 + Bcl2 + CD138 − DLBCL	14x2x5 cm	10%	IE	Thyroidectomy + cervical lymphadenectomy + Radiotherapy(30Gy)	2 years
3	CD20 + DLBCL	11x4x2 cm	20%	IE	Thyroidectomy + cervical lymphadenectomy	died immediately after surgery
4	DLBCLCD20 +	10,5 x5.6 x 6 cm	25%	IV	Thyroidectomy	died immediately after surgery
5	DLBCLCD20 +	5x2x2 cm	15%	IE	Lobo-isthmectomy + chemotherapy (CHOP) + radiotherapy 30Gy	3 years

DLBCL: diffuse large B-cell lymphoma; NA: not available

## Discussion

PLT is a rare tumor, histologically the most common subtype is a diffuse large B-cell lymphoma (DLBCL), which has a more aggressive clinical course and is usually diagnosed at a disseminated stage [5]. The pathogenesis of PTL remains obscure, but patients with DLBCL usually have a history of thyroid disease. It may be related to autoimmune disease or chronic inflammatory disease [6]. In our case one patient had a history of thyroid disease and hypothyroid.

Unlike the most common types of thyroid cancer, irradiation is not a factor in the disease, but long-standing Hashimoto’s thyroiditis is a frequent predisposing cause [3]. Currently the role of Hashimoto’s thyroiditis in the pathogenesis of PLT remains unclear, in fact some authors demonstrate that the risk of Hashimoto’s thyroiditis patients developing PTL is 40 to 80 times greater than that of the general population, while the others have found that it is associated with over 90% of the PTL [7]. Hypothyroidism has been observed in 30–40% of patients with thyroid lymphoma [6, 9]. In our case hypothyroidism was observed in 20% of cases.

PTL is more common in women, and is 2–8 times more common in women than in men [3]. It is extremely rare in the paediatric population, presenting more frequently in the seventh decade of life (median age 67). In contrast, secondary thyroid lymphoma occurs more frequently in middle-aged population (median age 42) [7, 9, 10]. However, our patients were older than those in the literature, with a mean age of 76.2 years, and the condition is more common in women.

The main clinical presentation of PTL is, enlarging neck mass painless and rapidly growing of goiter. It is sometimes accompanied by compressive symptoms such as dysphagia, difficulty swallowing, and pressure or shortness of breath. The results in the literature differ from those in our series, since all our patients had compressive symptoms. In this series, more patients are hypothyroid [7]. In contrast to the current study the majority of patients (four) were euthyroid.

On physical examination, the mass is often fixed to surrounding tissue and, in half of the cases, associated with unilateral or bilateral cervical lymph nodes. Our study showed similar results. Two patients had a mass fixed to muscle or to recurrent nerve. Cervical lymph nodes were found in two cases. In the series some systemic symptoms are reported (10%) such as fever and weight loss but these are uncommon [7, 12, 13]. The latency period before diagnosis is shorter than other thyroid cancers, it range from few days to 36 months, sometimes over a few weeks [3, 10].

The diagnosis of PTL can be established in the first instance by biochemistry; in 80% of cases, circulating antibodies to thyroid peroxidase are positive. 2-microglobulin levels have also been shown to be elevated in patients with non-Hodgkin’s lymphoma, and have been used to detect recurrence [8].

In particular, diagnosis depends primarily on imaging features and is confirmed by histopathological examination. Cervical ultrasonography has become the first standard imaging test for the diagnosis of thyroid mass, which presents in three forms: nodular, diffuse, or mixed. It also plays an important role in the initial diagnosis and follow-up [6].

Typically, PTL appears as a hypoechoic masses intermingled with echogenic structures with enhances posterior echoes and good vascularization. Unlike Hashimoto’s thyroiditis, PTL is rich in central-blood with high resistance artery blood flow [2, 6]. Ultrasonography is helpful for differentiating anaplastic thyroid carcinoma from other benign lesions such as subacute thyroiditis, or hemorrhage in a cyst or adenoma [8, 10]. Also it is particularly useful for establishing the diagnosis by guiding FNAC or core needle biopsies [13]. CT and MRI scans can confirm the presence of suspicious thyroid mass but are not recommended for the diagnosis [1].

Once the diagnosis of PTL has been suspected by clinical presentation and ultrasonography findings, the next step is histological confirmation by biopsy or FNAC which represent the technique of choice for pathological assessment of a thyroid mass [8].

The sensibility of FNAC is variable in the literature, in fact in the series by Agarwal *et al.* FNA accurately diagnosed PTL in only 60% of patients who were later found to have the malignancy, in other series from India (90%) of the cases of PTL were correctly diagnosed by FNAC, this variability may depend on the experience of the cytopathologists [13].

In our case, none of the patients underwent FNAC or biopsy, because of the emergency due to compressive symptoms and for the waiting time for cytological results (3 weeks in our institute), the diagnosis was made by surgical resection: total thyroidectomy or hemi –thyroidectomy, and the diagnosis was made after surgical excision.

Currently, the addition of other techniques like flow cytometry, or molecular biology such as polymerase chain reaction (PCR) have increased the sensitivity of FNAC by 97% and its specificity by 87% for the detection of B-cell lymphoma. The use of ultrasound to guide fine-needle aspiration also increases sensitivity and avoids biopsy of necrotic areas, while minimizing the risk of trauma to adjacent structures [10].

However, its role in the diagnosis is insufficient for differential diagnoses like Hashimoto’s thyroiditis, lymphocytic thyroiditis and anaplastic carcinoma thyroid, as well as for low grade lymphoma. For that usually we require a core needle biopsy or a surgical incisional biopsy or even thyroidectomy to make the diagnosis [8, 10, 14]. However, its role in diagnosis is insufficient for differential diagnoses such as Hashimoto’s thyroiditis, lymphocytic thyroiditis and anaplastic thyroid carcinoma, as well as for low-grade lymphomas. A needle biopsy or incisional surgical biopsy, or even a thyroidectomy, is usually required to establish the diagnosis.

If FNAC fails, and in cases where surgery is not indicated and to avoid unnecessary surgical resection, we perform a core biopsy as it provides more tissue than FNAC. It can be useful in distinguishing between PTL from others diagnoses, especially anaplastic carcinoma. The sensitivity of core biopsy can reach 95% and is useful for lymphoma subtyping [7, 8, 10, 11].

Currently, surgical open biopsy is only recommended when other techniques fail to achieve the diagnosis, or when the therapeutic strategy depends on the histological subtype, especially in our cases of the large B-cell lymphoma [7, 8].

It is very important to confirm the diagnosis from other differential diagnoses, notably anaplastic thyroid cancer and advanced medullary thyroid cancer, as the treatment and prognosis are very different [4, 9].

Staging procedures are the same as for other lymphoma subtypes, and are based first and foremost on physical examination, biochemical analysis including LDH and beta2-microglobulin, liver function test, bone marrow biopsy and whole-body CT or MRI as well as appropriate biopsies [1].

In aggressive varieties, PET/CT is useful for both staging and response to treatment. But it is not often performed due to lack of availability, so no patients in our series benefited from PET/CT. However Galium 67 Scintigraphy plays a role in detecting local recurrence. MRI is more sensitive than CT in detecting extrathyroidal extension [8, 10].

In previous series, most patients were diagnosed early at stage I, PLT can spread to the lymph nodes, but rarely bone marrow [3, 12]. In the present study all patients did not had any metastasis; According to Ann-Arbor Staging System the majority were staged as IE, one case as IIE and one case stage IV. although that all patients was diagnosed in stage of compressive symptoms. According to the Ann-Arbor staging system, the majority of patients were staged as IE, one case as IIE and one case as IV, although all patients were diagnosed at the compressive symptom stage.

Microscopically, DLBC appears as large, atypical lymphocytes with frequent mitosis. On immunohistochemical antibodies are used to detect antigens in thyroid tissue. The presence of antibodies to the B-cell antigens CD19 and CD20, for example, identifies a B-cell lineage in the lymphoid cells. Most DLBCL are Bcl-6-positive, and about half are Bcl-2-positive [12].

However, it is difficult to identify low-grade lymphomas due to their similarity to Hashimoto’s thyroiditis, which actually presents with a mixture of small and large lymphocytes. In some cases, additional tests are necessary such as immunophenotyping, flow cytometry, and PCR used to detect the immunoglobulin heavy chain gene [2].

The therapeutic approach in PTL has not been established due to the low frequency and the fact that most published studies involves only a limited number of patients. But it is generally based on established treatment for other extranodal non-Hodgkin’s lymphomas. It depends on histological finding and stage [12].

Currently, most authors propose a multidisciplinary approach because of the typically aggressive clinical course, based on surgery, radiotherapy, chemotherapy, or a combination of these and, recently, the introduction of the immunotherapy [6–8, 11]. Generally, surgery and radiotherapy are effective for local tumor control, however, chemotherapy is necessary at the stage of disseminated disease [2, 9]. PTL type DLBC is sensitive to radiotherapy and chemotherapy, especially the combination of CHOP chemotherapy (cyclophosphamide, doxorubicin, vincristine and prednisone) and the monoclonal antibody Rituximab, which targets CD20 CHOP [10].

Surgery once played a major role in the treatment of PTL, especially before to the introduction of FNA biopsy in the 1970s, as resection is no longer required for diagnosis. But currently, the role of surgical treatment in PTL remains controversial. When the diagnosis of DLBC is made by a non- invasive technique, thyroid resection may be difficult due to the inflammatory nature of thyroiditis and the infiltrating tumor, or extensive growth and possible extra-thyroidal extension [2, 13].

Recently, some authors think that the role of surgery is limited and have a limited benefit in order to preserve the function of the thyroid gland; in fact aggressive surgery is not necessary because it has many risks include damage to the recurrent laryngeal nerves, the trachea, the esophagus and large vessels. Also a Turkish series found that there is no significant differences in survival rates among patients who underwent surgery followed radiation therapy and combinations of radiation and chemotherapy without extensive surgery [1, 14].

The Mayo clinic, in its largest study including 62 patients with PTL, found that the combination of total thyroidectomy with adjuvant radiotherapy did not demonstrate an increase in survival compared to a biopsy associated to radiotherapy in stages IE or IIE, on the other hand surgery may causes morbidities without an improvement in survival rate [15].

In some cases, surgery may also be used to obtain a diagnosis when other non-invasive techniques have failed. In many cases, PTL are still diagnosed on definitive histopathological examination of a surgical specimen, when surgery was indicated because of suspected thyroid cancer [3, 12].

Also others contended that surgery favorably influenced the outcome in patients with PTL in certain small case series [5]. Also Alfonso *et al.* found that surgical debulking have a role in symptom control, especially in the case of compressive symptoms to relieve pain, in fact, in his series 4 patients from 7 underwent surgery alone or followed with radiotherapy [10].

However, surgery is helpful, especially for compressive symptoms, as a palliative treatment, and for the protection of the airway [10]. The use of less invasive techniques like tracheostomy and corticosteroids to secure the airway, followed by definitive treatment, is better than to use of invasive surgery [12].

In the present study, all patients had compressive symptoms and ultrasound did not confirm the type of thyroid cancer. Due to the high frequency of papillary cancer in our country, we preferred surgery in the first instance for diagnosis and to alleviate the compression.

Thyroid lymphomas have been shown to be both chemosensitive and radiosensitive. Some authors have indicated local radiation only if the malignancy is completely intra-thyroidal, and can be added according to tumor size and stage [12].

Currently, there are two radiotherapy regimens that can be administrated to patient with PTL: “Involved-field radiotherapy” that includes only the thyroid bed and cervical lymph nodes only, and “extended-field radiotherapy” that also includes mediastinal nodes and sometimes the axillary lymph nodes. The second regime seems to be superior to reduce local recurrence (52% vs 27%). It should be given in a total dose of 40 Gy to the neck and mediastinum [8].

Radiotherapy alone should only be used when the disease is confined to the thyroid (Stage 1 Disease). Adjuvant radiotherapy after surgical resection has also proved highly beneficial. In fact the addition of the radiotherapy brings a survival benefit of 1.5 years. However it must be combined with chemotherapy beyond the first stage when the disease extends outside the thyroid, in advanced stage chemotherapy is beneficial to reduce tumor size [3, 12].

A recent study in Endocrinology published in March 2020 suggested that the use of chemotherapy is beneficial even in localized stages (I and II) for small tumors less than 5 cm but with limited courses (three) [3]. Radiation therapy is used to consolidate the response to CHOP for those receiving only three courses. Currently, rituximab is given as part of the CHOP regimen (R-CHOP) [8].

Typically, it is administered after the third cycle of CHOP chemotherapy. The use of the association decrease distant recurrence [8]. A recent study showed that the use of chemotherapy alone is not recommended because it is inferior than the association [8].

The protocol of chemotherapy consists of three to six cycles of the CHOP regimen (doxorubicin 50 mg/m^2^, cyclophosphamide 750 mg/m^2^, vincristine 1.4 mg/m^2^, prednisone 40 mg/ m^2^) and lasts for six months. The mainstay of chemotherapy after complete remission is not uncommon [11]. This protocol may not be suitable for patients in poor general health conditions [3].

Recent study demonstrated that surgery is used just for diagnosis, the ideal treatment of PTL and especially DLBC is the combination of chemotherapy and radiotherapy even at early stage [1]. This combination is associated with a significantly lower risk of spread disease in locally advanced tumors compared to the use alone of radiotherapy [3, 7]. However Alfonso *et al.* think that radiotherapy is not necessary in almost of cases because chemotherapy give a good results, it has been used in patients who cannot received surgery or chemotherapy [10]. Chen *et al.* found that combined chemotherapy regimen is the most common choice for PLT, The aim of combination is to reduce the incidence of distant recurrence [5].

In their study Onal *et al.* confirmed the survival advantage of multimodal therapy. Overall Survival was 91% for patients receiving combined modality therapy, compared with 57% for those receiving chemotherapy alone (*p* = 0.01) and 69% for those receiving radiotherapy alone (*p* = 0.03) [16].

Also Mian *et al.* found that DLBCL patients who underwent the combination of chemotherapy and radiotherapy had a statistically significant increase in PFS than those who received single therapy [17].

Recently, the introduction of Rituximab, a monoclonal antibody anti-CD20, represented a significant advance in the treatment of DBCL, this treatment should be combinated with chemotherapy based on CHOP or CVP (cyclophosphamide, vincristine, and prednisone), and this protocol improves disease-free and overall survival. It can also result in a complete remission without disease recurrence 16–25 months after therapy completion [7, 8, 10, 13]. The association may causes neutropenic fever so the clinicians should be careful about this clinical manifestation [18, 19]. The maintenance therapy with Rituximab after chemotherapy improved significantly progression-free survival in advanced-stage [8].

In conclusion, in localized disease (Stage I or II), the treatment based on chemotherapy (CHOP) and local radiotherapy, however surgery is often reserved for diagnosis and to relief air way compression [11, 13]. But in some patients under 60 years and without any adverse prognostic factors CHOP alone may be proposed [9].

However, in Disseminated, aggressive disease: patients should be treated with radiotherapy and multi agent chemotherapy 6 courses of CHOP and Rituximab [11].

In the present study two patients died immediately post operative so they did not received any adjuvant therapy, one patient had stage IIE he underwent hemi-thyroidectomy followed by chemotherapy (CHOP) and Ritiximab, the two others patients were classified IE they underwent surgery followed by radiotherapy 30 Gy.

PTL have a favorable outcome with appropriate therapy, but DLBC has poor prognosis due to some factors such as rapid clinical growth, and the presence of B symptoms, dysphagia, or stridor, also advanced stage of the tumour, in fact the 5 survival rates for stage I ranging 70 to 80 percent increasing to 30–40% for stage III and IV. As well as large size (> 10 cm), the extension outside of the thyroid especially to the mediastinum and lymph node metastases [1, 3, 8, 11].

PTL have a risk of both locoregional and distant relapse sow use combined modality approaches that use chemotherapy with radiotherapy [13]. In the current study, the average of follow-up was 17 months (0 to 36 months), two patients were classified IE died from respiratory complication and the two others were flowed up and none of them developed local or distant recurrence.

## Conclusion

Primary thyroid lymphoma is rare. Presenting with an enlarging goiter and associated compressive symptoms, ultrasound guided FNA is the first step of suspicion of lymphoma. Treatment and prognosis are dependent on accurate histological classification. Multimodal treatment with rituximab, combination chemotherapy, and local radiotherapy provides the highest over survival rates.

## Data Availability

Data supporting our findings were taken from the patient’s folders.
